# Development and Evaluation of Interprofessional High-Fidelity Simulation Course on Medication Therapy Consultation for German Pharmacy and Medical Students—A Randomized Controlled Study

**DOI:** 10.3390/pharmacy12040128

**Published:** 2024-08-21

**Authors:** Ahmed Reda Sharkas, Bushra Ali Sherazi, Shahzad Ahmad Sayyed, Florian Kinny, Melina Steichert, Holger Schwender, Stephanie Laeer

**Affiliations:** 1 Institute of Clinical Pharmacy and Pharmacotherapy, Heinrich Heine University Duesseldorf, Universitaetsstrasse 1, 40225 Duesseldorf, Germany; bushra.ali.sherazi@hhu.de (B.A.S.); shahzad.sayyed@hhu.de (S.A.S.); melina.steichert@hhu.de (M.S.); stephanie.laeer@uni-duesseldorf.de (S.L.); 2 Institute of Pharmacy, Faculty of Pharmaceutical and Allied Health Sciences, Lahore College for Women University, Lahore 54000, Pakistan; 3 Mathematical Institute, Heinrich Heine University Duesseldorf, Universitaetsstrasse 1, 40225 Duesseldorf, Germany

**Keywords:** interprofessional education, pharmacy students, medical students, high-fidelity simulation, interprofessional training, pharmacy education

## Abstract

Recently, there has been a remarkable move towards interprofessional collaboration in response to the COVID-19 pandemic and the care of comorbidities. In Germany, there has been a gradual increase in interprofessional learning in medical and pharmacy education, aiming to enhance patient care. To adapt the pharmacy curriculum for collaborative practice between pharmacy and medical students, we developed an immersive interprofessional collaboration course for pharmacy students using adult and pediatric high-fidelity simulators (HFS) to assess and train medication consultation skills. In a randomized controlled trial, we investigated whether interprofessional training between pharmacy and medical students results in differences in pharmacy students’ performance of medication therapy consultation compared to the case of mono-professional training of pharmacy students only. Before and after inter/mono-professional training, each pharmacy student performed an objective structured clinical examination (OSCE) and completed a self-assessment questionnaire. Additionally, an attitude survey towards interprofessional learning was completed by pharmacy and medical students at the end of the training. As expected, interprofessional as well as mono-professional training showed a statistically significant increase in medication consultation skills. Of importance, the performance in the interprofessional training group was significantly better than in the mono-professional group, particularly in drug therapy counselling and consultation behaviors. There was a significant difference between the intervention and control groups in self-assessment scores, and all study participants had positive attitudes toward interprofessional collaboration and training. Therefore, interprofessional training using HFS has been shown to appropriately train pharmacy students for collaborative practice and consultation skills.

## 1. Introduction

Interprofessional collaboration (IPC) is the keystone of training healthcare professionals to provide patient-centered care [1,2], involving multiple professions working collaboratively to improve health outcomes [3]. In addition to its beneficial impact on healthcare practitioners and multimorbid patients, IPC provides non-medical benefits, such as lower healthcare costs and less workload for physicians [4]. Conversely, less collaborative patient care can lead to undesirable effects and hospitalization [5]. Around 35% of the European population suffers from chronic diseases [2], and up to 40% of hospitalized patients are over 65 years of age [6]. In Germany, about 62% of patients aged 65 or older suffer from multimorbidity [7]. Furthermore, polypharmacy affects 42% of geriatrics, which leads to adverse effects and hospitalizations, and 5% of hospital admissions are related to unwanted drug effects [8,9]. Thus, medication therapy management forms an essential part of the healthcare system [10]. Collaboration between physicians and pharmacists improves patient treatment, where the pharmacist can optimize drug therapy and minimize adverse effects [5,11].

Interprofessional education (IPE) is recognized as the best practice for health professionals [12], developed by institutions worldwide and included in accreditation standards, which enables two or more professions to learn from, about, and with each other to collaborate in the delivery of patient care [13]. The World Health Organization (WHO) and the International Pharmaceutical Federation (FIP) provide strategies for implementing IPE models, focusing on the collaboration of pharmacists with other healthcare providers [3]. Furthermore, the Committee of the German Association for Medical Education (GMA) advocates developing IPE models to promote cooperation among professionals, through the integration of interprofessional programs into academic curricula and the incorporation of assessment methods for interprofessional competencies [14,15]. IPE activities include hands-on clinical training, such as medication reviews and clinical rotations, as well as simulation-based learning like simulated hospital wards. Additionally, IPE lectures and workshops cover topics such as medication safety, patient-centered care, communication skills, and collaborative practice [16,17,18]. Many collaborative activities traditionally place students in multidisciplinary clinical settings [19], recognizing the crucial role of consultation and communication skills in IPC in which the role of the pharmacist in the interprofessional team is essential in providing pharmaceutical care to patients and managing medication-related problems [20].

As a result, several countries, such as the United States, Canada, and the United Kingdom, proliferate IPE models into pharmacy curricula [17,21,22]. In 2011, the United States adopted the Interprofessional Collaborative Education Framework (IPEC) to enhance interprofessional learning, leading that IPE became a central to PharmD programs to prepare graduates for collaborative practice. Furthermore, Canada extended interprofessional activities across healthcare curricula, for instance, a developed interprofessional curricular framework to teach pharmacy students deprescribing skills, particularly for managing complex patient cases [13,23,24]. In the United Kingdom, collaboration among health professionals has expanded the pharmacist’s role to include medication review [5]. Additionally, various universities use different IPE models, like the Leicester Model, which focuses on the knowledge and attitudes needed for interprofessional practice through face-to-face activities, as well as virtual IPE such as telehealth projects, which facilitate interprofessional learning remotely [25]. Switzerland introduced a national framework for IPE through legislative changes in 2021. In Germany, IPE was introduced after being overlooked for several years; in 2015, the Robert Bosch Foundation launched several projects to support interprofessional learning, later with the implementation of interprofessional clinical training wards in 2017. Despite the importance of these projects, their embedding in the curriculum has not yet been well addressed. Recently, German healthcare education regulations have directed an interprofessional approach to nursing training and medical curricula [15,26,27]. Additionally, the Federal Association of Pharmacy Students (BPhD) and the Federal Representation of Medical Students (bvmd) advocate further cooperation between physicians and pharmacists, aiming to improve patient medication therapy [28]. For instance, few interprofessional projects in Germany were conducted during the COVID-19 pandemic [8,29,30,31]. However, a training model for physician–pharmacist collaboration with objective assessment tools to measure learning outcomes has not yet been introduced in Germany [15,32]. Despite efforts to promote IPE for pharmacy students, the combination of theoretical input and practical work remains a concern in the development of IPE curriculum, alongside barriers such as insufficient curriculum space and funding constraints [33,34].

Healthcare education incorporates simulation as a formative teaching and assessment strategy [35], recognizing its importance in improving collaboration and communication skills [36]. Simulation methods are incorporated into medical and nursing education programs [36,37], as well as pharmacy curricula [38,39]. Simulation methods are classified according to their level of physiological function including high-fidelity simulation (HFS), low-fidelity simulation (LFS), and standardized patients. HFS enables health profession students to learn and practice their skills in a way that mimics professional practice and allows students to experience the consequences of their decisions in a safe environment [40]. Consequently, HFS used to enhance students’ performance and attitudes to IPE [39]. Moreover, the Objective Structured Clinical Examination (OSCE) is a standard assessment method for clinical competency in healthcare education particularly the evaluation of consultation skills [41,42,43].

Upon reviewing the literature, the authors found that previous studies demonstrated the benefits of interprofessional education among healthcare professionals. However, these studies employed observational and hypothesis-generating approaches or quasi-experimental designs without random assignment [44,45,46,47]. Moreover, there is a limited number of randomized controlled trials (RCTs) that focus on the comparative effectiveness of interprofessional groups to mono-professional control groups (e.g., medical or nursing students [48,49,50]), with a lack of such studies specifically using pharmacy students as controls. Additionally, we found no studies on interprofessional simulation-based OSCE for pharmacy students at German universities that assess the effectiveness of interprofessional training compared to traditional mono-professional approaches.

Therefore, we conducted a randomized controlled study to investigate whether interprofessional training, including medical and pharmacy students in terms of medication therapy consultation, compared to a mono-professional training containing pharmacy students alone, results in differences in the performance and self-assessment of pharmacy participants. The primary objective of this study is to develop an immersive interprofessional training course using high-fidelity patient simulators of different age groups to train collaborative and consultation skills, including acute care scenarios of adults and children. The secondary objective is to survey the participants’ attitudes towards interprofessional learning.

## 2. Materials and Methods

### 2.1. Study Design and Randomization Procedure

**Figure 1:**
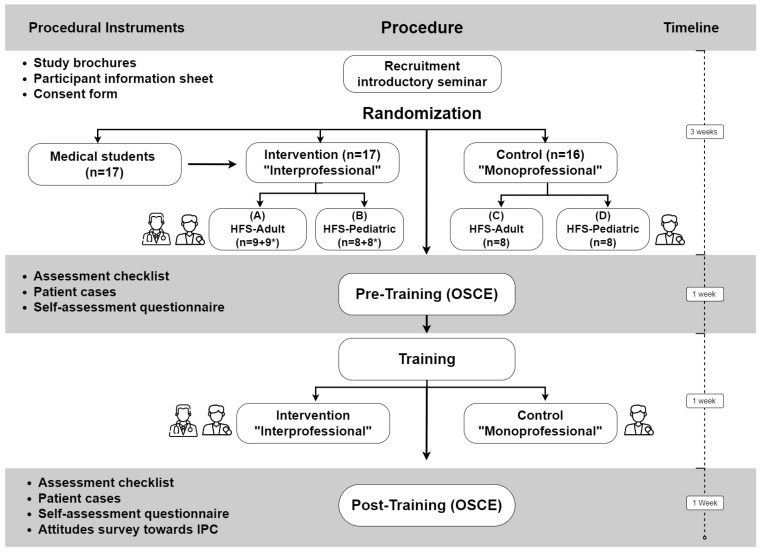
Flowchart of the randomized controlled study. OSCE = objective structured clinical examination. HFS = high-fidelity simulation. The flowchart was designed using draw.io [52]. (*) = number of medical students. IPC = interprofessional collaboration.

A randomized controlled trial was carried out with pharmacy and medical students to investigate the impact of interprofessional training on medication consultation skills using adult and pediatric scenarios with HFS, compared to a mono-professional training. We assessed and compared the pre-training baseline performance with post-training performance in a pre–post design. The study was conducted in German language from November until December 2023 as part of the clinical pharmacy course at Heinrich Heine University in Duesseldorf, following the approval of this study by the responsible ethics committee (Nr. 2023-2593). In November 2023, fourth-year pharmacy and fifth-year medical students were invited to participate in the study. After completing the informed participation procedure by voluntarily signing the consent form, a total of 33 pharmacy and 17 medical participants were randomized into intervention groups or control groups using Microsoft Excel 365 (Version 2403) [51]. To protect the confidentiality of participants’ personal data, we assigned pseudonymized codes to all participants using the function “RAND” in Microsoft Excel. Additionally, the pharmacy participants were randomly allocated into four groups: A and B were intervention groups with adult and pediatric patient simulators, respectively, while C and D were control groups with the same simulators. Medical participants were randomly assigned to groups A and B only, as shown in Figure 1, illustrating the flowchart of the study procedure and timeline.

### 2.2. Study Procedure

Firstly, as shown in Figure 1, we recruited the students by distributing brochures containing all relevant information about the study, followed by an introductory seminar for both medical and pharmacy students within 3 weeks, in which they were informed in detail about the study. The seminar included background information about the interprofessional collaboration and education status globally and the role of pharmacists in interprofessional team, in addition to a brief discussion about the current status and national initiatives in Germany for interprofessional learning projects. Then, participant information sheets and consent forms were handed out to the prospective participants. Upon voluntarily signing the consent forms, the students became eligible to participate in the study. Afterwards, the pharmacy participants completed a pre-training OSCE and a self-assessment questionnaire within one week. Subsequently, for an additional week, we conducted interprofessional and mono-professional training including HFS, which included adult and pediatric acute care scenarios for the four groups. The control groups received mono-professional training, including pharmacy students only, while in the intervention groups pharmacy and medical students trained collaboratively together. All participants completed an attitudes survey towards interprofessional learning at the end of study trainings. Finally, the pharmacy participants completed a post-training OSCE and another identical self-assessment questionnaire.

### 2.3. Objective Structured Clinical Examination

**Figure 2:**
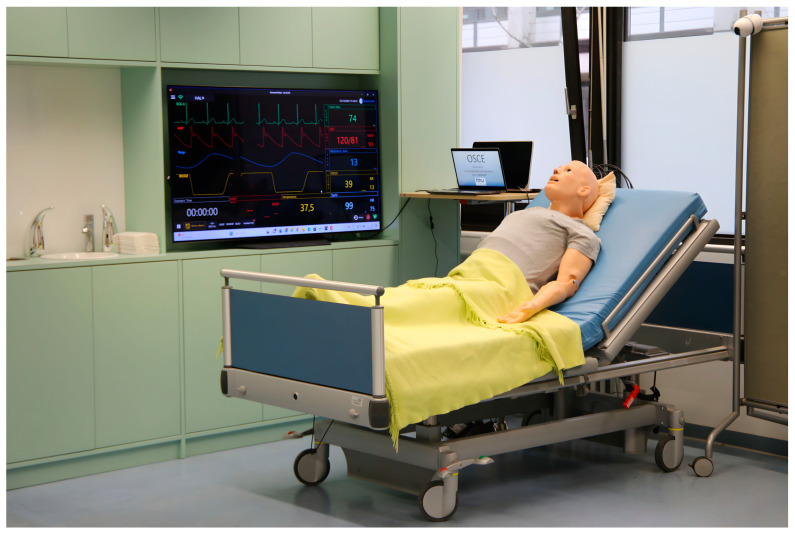
Inpatient care—simulated consultation room including high-fidelity simulator and portable camera system.

In the pre- and post-OSCEs, pharmacy students were assessed individually for medication consultation skills to measure any possible differences. Two faculty members prepared four OSCE cases for adults and four for pediatrics, and two other faculty members reviewed the cases; these cases were discussed in detail with other study staff during meetings. Additionally, inpatient care—a simulated consultation room—was set up with all necessary instruments for the OSCE scenario (Figure 2), in which each pharmacy participant performed a medication consultation with either adult or pediatric patient simulator (HAL^®^ S1000, S3005 Gaumard). The simulators used in the study were obtained from Mefina Medical GmbH & Co. KG, located in Erkrath, Germany.

The medication consultation involved performing anamnesis, including evaluating current patient symptoms; interpreting lab results and vital signs; managing medication-related problems; and providing medication counselling. Two faculty members assessed the OSCEs as examiners. Prior to the pre-OSCE, participants were briefed on the overall procedure and their individual time frame for each OSCE, which lasted 30 min. For adult pre- and post-OSCE, one examiner assessed both control and intervention groups. Similarly, for pediatric OSCEs, the second examiner assessed both groups. Based on the participants’ consent, selected OSCEs were recorded to review the quality of assessments.

### 2.4. Training Sessions

**Figure 3:**
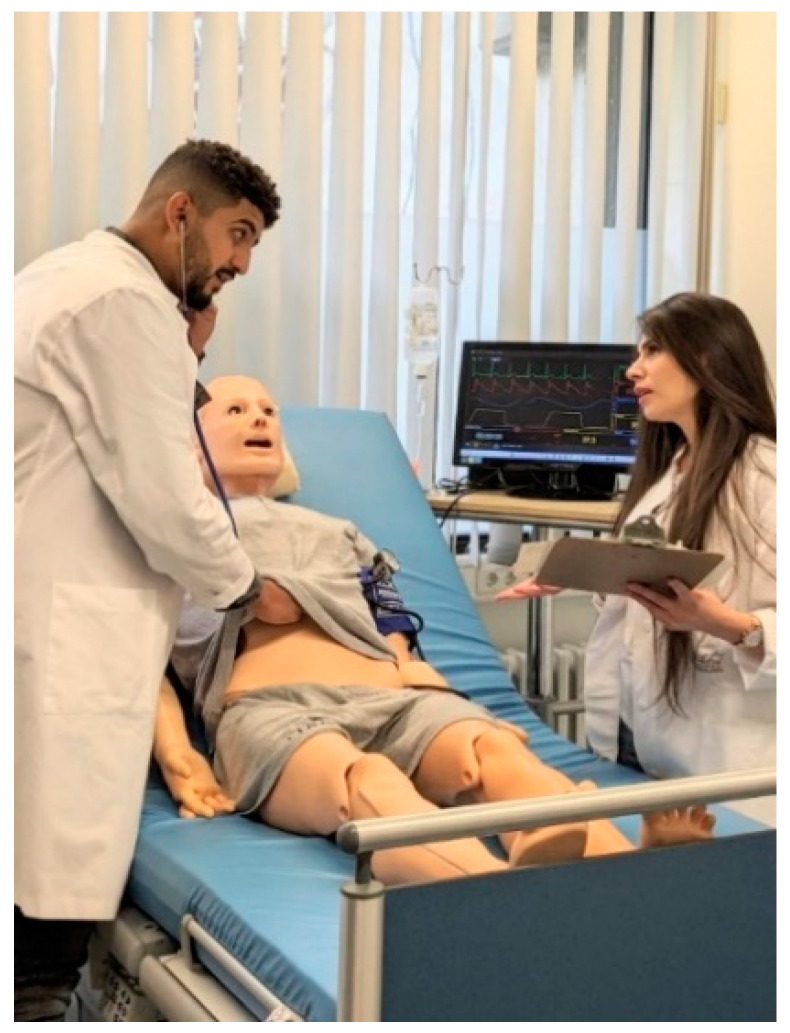
Medication consultation between medical and pharmacy student using high-fidelity simulator displaying symptoms and vital signs.

The training session started with a theoretical session (1 h) separately for the intervention (A, B) and control groups (C, D), followed by a practical session (4 h) for each study group (Figure 3). Both groups received the same theoretical training on the different models and styles of medical consultation, roles and responsibilities of physicians and pharmacists in an interprofessional team, and the Medication-Related Consultation Framework (MRCF) and its application [53].

The practical sessions included eight training cases for adults and eight for pediatrics, focusing on taking patient’s medical history, and applying patient-centered care techniques, providing medication counselling. Additionally, all participants were trained on consultation and communication skills, as well as recognizing and managing potential medication-related problems via either an adult or pediatric patient simulator; each intervention group comprised one medical and one pharmacy student that worked collaboratively to manage acute scenarios, while each control group consisted of only two pharmacy students. All participants spoke directly to the HFS via an in-built microphone, and two faculty members either remotely controlled the simulator or acted as a family member of the pediatric patient and responded to the participants. In addition, various acute scenarios were simulated by changing the simulator’s physiological symptoms and vital parameters on a displayed monitor through a customized simulator software program.

### 2.5. Instruments

#### 2.5.1. High-Fidelity Simulators

**Figure 4:**
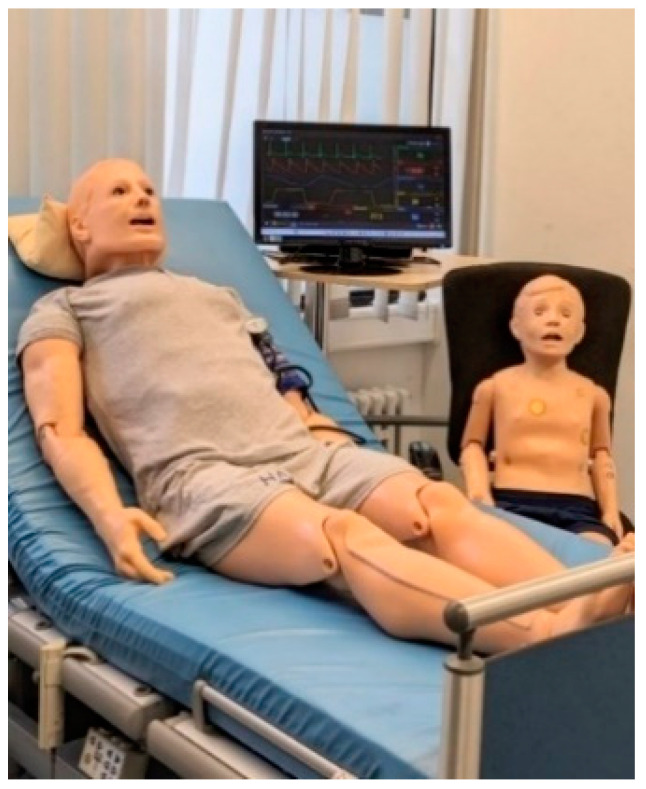
Adult and child high-fidelity simulators with software-displayed vital parameters.

We used HFS of different age groups as shown in Figure 4 (adult HAL^®^ S1000, pediatric S3005 Gaumard) to train consultation and communication skills. All participants practiced medication consultation and were exposed to various scenarios, including adverse symptoms associated with medication-related issues. HFS enable realistic inpatient care consultation through physiological responses to the participants’ interventions; the participants could observe the adverse symptoms and the vital signs either physically on the simulator or on a displayed monitor, then perform the necessary measures. Customized software controls both simulators including a built-in microphone, which allows the instructor to communicate simultaneously with the participant and provides demanding intervention tailored to the patient scenario. Furthermore, HFS possess several controllable features, including physiological parameters, such as blood pressure, respiratory/heart rate, and oxygen saturation; other physical attributes, such as palpable pulse, heart and lung sounds, chest and abdominal movements, in addition to programmable eye blinking rate, cyanosis, and simulated seizures with selectable intensities [54,55].

#### 2.5.2. Nordic RecMobile System

Nordic RecMobile, a portable recording system, allows instructors to remotely observe student performance and adjust the scenario flow as needed, and provide feedback to participants based on their actions during simulation training [56].

#### 2.5.3. Medicheck

Within the setting of a professional practice, participants had access to Medicheck education, a digital medication review tool, to assist in the medication review process, such as highlighting potential drug interactions [57]. As part of the clinical pharmacy course at the university, pharmacy students attend seminars on how to use Medicheck [58]. These seminars enabled pharmacy participants to support medical participants to become familiar with the Medicheck features during the training. In a specific part in the OSCEs, pharmacy participants obtained patient case information through Medicheck for medication reviews. Afterwards, they engaged in a consultation with the patient simulator with the pre-existing information from Medicheck. While interacting with the HFS, new information regarding medication-related problems is revealed based on each patient’s case. Participants interpreted and managed the patient’s case using a combination of pre-existing information from Medicheck and information gained directly from the patient interaction during consultation.

#### 2.5.4. Cases for OSCEs

For the pre- and post OSCE, four OSCE cases for adults and four for pediatrics with acute care scenarios were prepared and reviewed. The cases cover diseases in adults and pediatrics such as hypertension, diabetes mellitus, dyslipidemia, bronchial asthma, Attention-Deficit/Hyperactivity Disorder (ADHD), and postmenopausal osteoporosis, in addition to various medication-related problems, such as drug–drug interactions, adverse events induced by over the-counter (OTC) drugs, inappropriate administration time or technique, and medication-induced deficiencies in certain vitamins and electrolytes.

All OSCE cases have the same pattern, in which a patient is admitted to the inpatient room for consultation due to an acute illness. For each case, we prepared and provided participants with a patient history sheet containing all necessary information, including the simulated medication package. For the simulation of patient roles, two faculty members were thoroughly briefed and provided with all the necessary information, including the patient scenario guide and script for each case. Additionally, they were uniformly trained through roleplay patient scenarios, either by simulating an adult patient using a simulator or by acting as a family member of the pediatric patient. Furthermore, we prepared specific checklists that were the same in terms of structure and scoring but different in the content of assessment items based on each OSCE case.

#### 2.5.5. Assessment Checklist

We adopted an assessment checklist from the Medication-Related Consultation Framework (MRCF), which is designed to teach and evaluate medication-related consultation skills [59]. However, adjustments were made to tailor the checklist specifically to the patient cases and to modify the checklist for objective assessment purposes. The checklist follows the general structure of MRCF and includes relevant items for the OSCE cases. The checklist contains 35 points in total. The scoring of the participant relied on executing each subitem specified in the checklist; each participant receives one point if fulfilled and zero points if not. The checklist consists of five sections. Each section is classified into subcategories and subitems with different total scores. Section 1 is named “patent information” with a total of 5 points, which outlines the initial steps of patient engagement in the consultation process. It involves the participant introducing themselves and confirming the patient’s identity by asking for their full name and date of birth. The purpose of the consultation is then explained to the patient, and the patient’s consent to proceed is obtained before continuing further. Section 2 is named “anamnesis” with a total of 8 points, which involves the participant gathering the patient’s medical history. This includes inquiries about the patient’s main complaints, the onset and duration of symptoms, associated symptoms, medication adherence, current medications (including any medication not listed in the patient history sheet), abnormal lab values or vital signs, and specific information regarding the patient’s personal and social history such as tobacco use, alcohol consumption, and dietary habits.

Section 3 is titled “actions and solutions “with a total of 10 points, which involves identifying and addressing the existing medication-related problems. It begins with identifying potential drug–drug interactions and providing recommendations for optimization. The participant also addresses medication-related problems by recognizing their occurrence, explaining underlying reasons, and providing optimization suggestions. Additionally, the participant asks about patient understanding of medication administration technique or timing, recognizes and addresses the existing inappropriate administration technique or timing, and provides further tailored recommendations depending on each case to enhance patient’s medication therapy. Section 4 is titled “closing” with a total of 3 points, which involves instructing the patient to report all medications to the physician, offering the patient to ask further questions regarding the problems discussed during the consultation, and expressing gratitude for the patient’s collaboration while requesting a follow-up. Finally, Section 5 is named “consultation behaviors” and focuses on encouraging patient involvement during case scenarios with a total of 9 points. This section involves the participant listening attentively to the patient’s concerns and questions without interrupting, responding informatively and reassuringly to patient concerns, avoiding medical jargon, maintaining confidence and control over the consultation by avoiding any irrelevant discussions, employing effective non-verbal communication including appropriate gestures, and managing consultation time effectively. The template OSCE checklist for the patient cases is attached in Appendix A.

#### 2.5.6. Self-Assessment Questionnaire

We developed a questionnaire to investigate pharmacy participants’ self-assessment of their competence in consultation and communication skills related to medication therapy, as well as interprofessional and simulation learning. The survey comprised 12 statements with a 5-point Likert scale, where 1 represented ‘strongly disagree’ and 5 represented ‘strongly agree’. Statements 1–6 address consultation and communication skills for medication consultation, statements 7–8 are related to interprofessional learning with other health profession students, and statements 9–12 concern the use of HFS in interprofessional and medication consultation in clinical pharmacy education. The self-assessment questionnaire was given before pre-OSCE and after post-OSCE.

#### 2.5.7. Attitudes Survey toward Interprofessional Learning

An attitudes survey was developed towards interprofessional learning among medical and pharmacy participants, which is inspired by two validated surveys: the Scale of Attitudes Toward Physician-Pharmacist Collaboration (SATP2C) and the Student Perceptions of Physician-Pharmacist Interprofessional Clinical Education (SPICE) [60,61]. The objective is to explore attitudes towards interprofessional learning among medical and pharmacy participants. The survey focuses on three aspects: collaborative medication management (4 items), roles and responsibilities in interprofessional team (2 items), and interprofessional training (4 items). Two items (Q6, Q10) are inspired by SATP2C, while other items (Q8, Q9) are inspired by SPICE; these items were modified to address the different aspects of the survey in the context of the study training objectives. The survey comprised 10 statements in total, with a 5-point Likert scale, where 1 represented ‘strongly disagree’ and 5 represented ‘strongly agree’.

### 2.6. Statistical Methods

We assessed the effect of the interprofessional training on medication consultation compared to the mono-professional training via OSCEs. The results are presented as OSCE checklist score points between pre- and post-OSCEs, as well as the intervention and control groups. Non-parametric tests were used to compare and assess the score points of the intervention and control groups; in particular, a one-sided paired Wilcoxon signed-rank test with a significance level of alpha = 0.05 was used to measure the effect of the OSCE training using HFS for the respective groups. Additionally, a one-sided Mann–Whitney test with a significance level of alpha = 0.05 was performed to determine the difference between the intervention and the control groups in terms of pre- and post-OSCEs. Furthermore, for data calculations, Microsoft Excel 365 [51] was used and OriginPro 2021 [62] was used for statistical analysis.

## 3. Results

### 3.1. Participant Characteristics

After voluntarily signing an informed consent form, 33 fourth-year pharmacy students and 17 fifth-year medical students participated in the study. Students were asked to indicate whether they had experience in drug therapy consultation (e.g., outpatient or inpatient hospitals), such as taking patient medication history and addressing patient medication therapy problems. Table 1 and Table 2 present the characteristics of the pharmacy and medical participants for both the intervention and control groups.

### 3.2. Checklist Scores for OSCEs

Pharmacy participants’ performance during the OSCEs was assessed using an assessment checklist. The checklist scores reflect the participants’ ability to successfully perform medication consultation, from engaging the patient in the consultation process and performing anamnesis to managing medication-related problems. The total points for each OSCE case for either adult or pediatric patients are 35 points.

#### 3.2.1. Checklist Scores Control and Intervention Groups (HFS-Adult)

**Figure 5:**
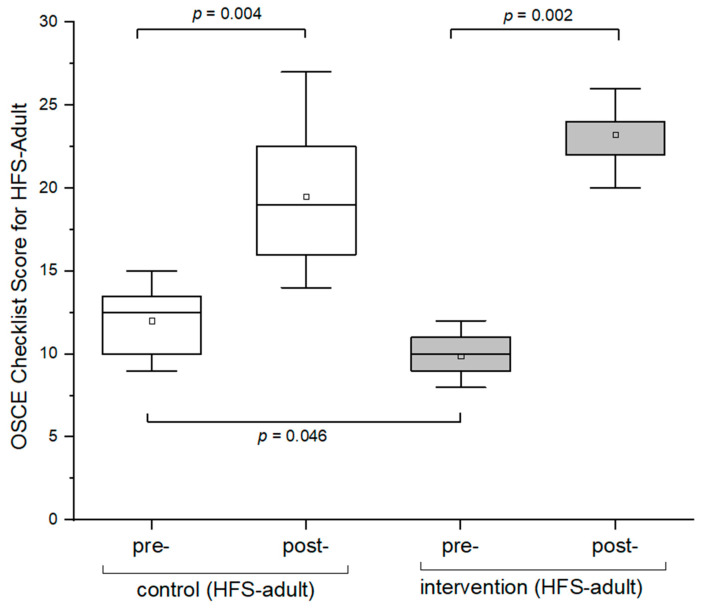
Box plots of OSCE checklist scores between pre- and post-OSCEs (HFS-adult). A one-sided paired Wilcoxon signed-rank test with a significance level of alpha = 0.05 was used to compare OSCE scores between pre- and post-OSCE for the control and intervention groups. OSCE = Objective Structured Clinical Examination. HFS = high-fidelity simulator. The hollow blocks (□) represent the means of the overall scores of pre- and post-OSCEs for the respective groups.

**Figure 6:**
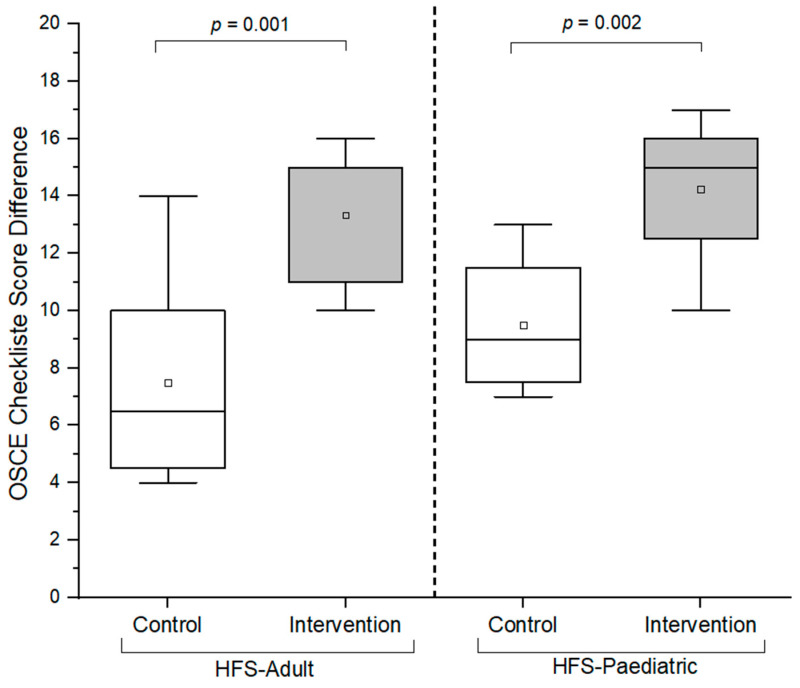
Box plots of OSCE checklist score differences between the control and intervention groups (HFS-adult and HFS-pediatric). A one-sided Mann–Whitney test with a significance level of alpha = 0.05 was used to compare OSCE scores between the control and intervention groups. OSCE = Objective Structured Clinical Examination. HFS = high-fidelity simulator. The hollow blocks (□) represent the means of the overall score differences for the respective groups.

At baseline, the OSCE scores were found to significantly differ between the control and the intervention group, as shown in Figure 5 (*p* = 0.046). Additionally, both control and intervention groups a showed significant improvement in their performance after the OSCE training (intervention group: *p* < 0.05; control group: *p* < 0.05; Figure 5). Moreover, the intervention group demonstrated a significant improvement in their overall checklist scores compared to the control group (*p* < 0.05; Figure 6; Appendix B; Table A1).

**Figure 7:**
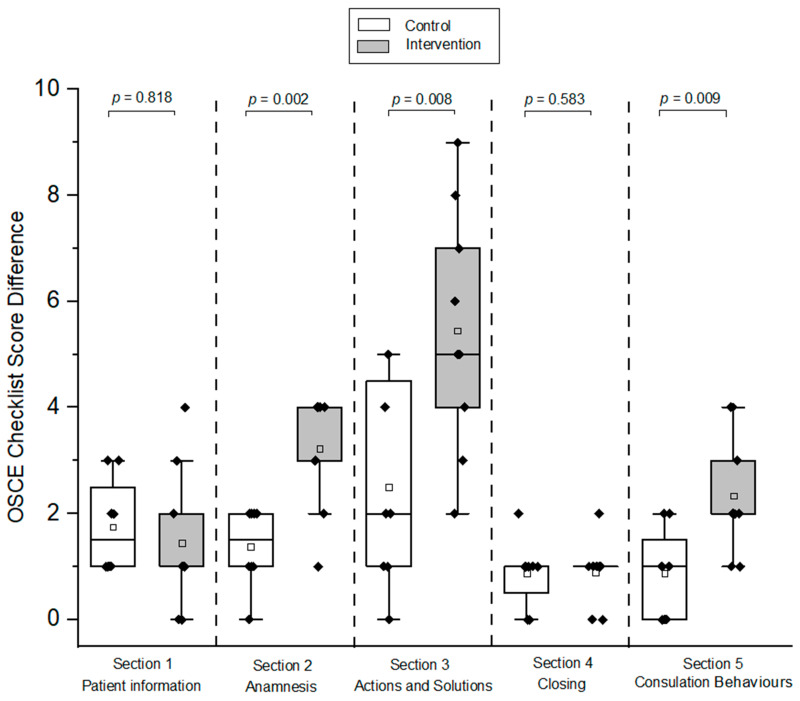
Box plots of OSCE checklist score differences for each section between pre- and post-OSCE for intervention and control groups (HFS-adult). A one-sided Mann–Whitney test with a significance level of alpha = 0.05 was used to compare OSCE scores between the respective groups. The black diamonds (♦) indicate the score difference of each participant of post- and pre-OSCE. OSCE = Objective Structured Clinical Examination. HFS = high-fidelity simulator. The hollow blocks (□) represent the means of score differences in each OSCE checklist section for the respective groups.

The intervention group showed a significant improvement in Sections 2, 3, and 5 compared to the control group, as shown in Figure 7 (*p* < 0.05); however, there was no significant difference for Sections 1 and 4 related to patient information and consultation closing, respectively (*p* = 0.818 for Section 1; *p* = 0.583 for Section 4; Figure 7).

#### 3.2.2. Checklist Scores Control and Intervention Groups (HFS-Pediatric)

**Figure 8:**
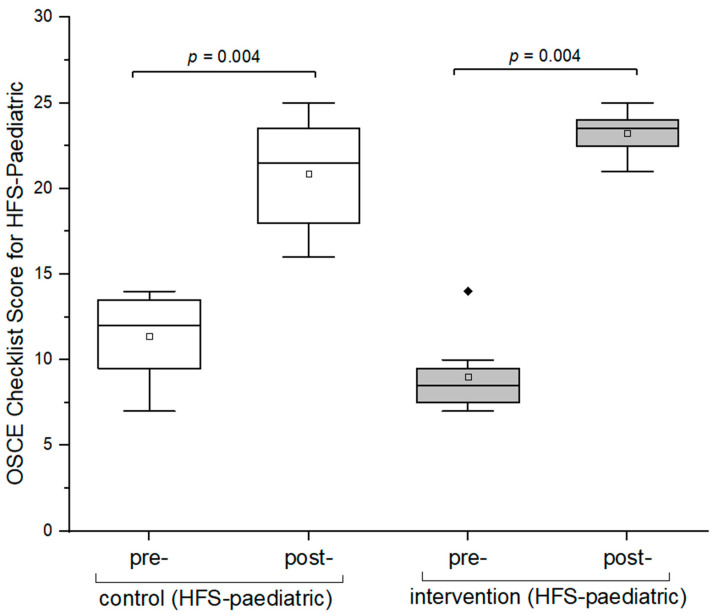
Box plots of OSCE checklist scores between pre- and post-OSCEs (HFS-pediatric). A one-sided paired Wilcoxon signed-rank test with a significance level of alpha = 0.05 was used to compare OSCE scores between pre- and post-OSCE for the control and intervention groups. The black diamonds (♦) indicate the outliers. OSCE = Objective Structured Clinical Examination. HFS = high-fidelity simulator. The hollow blocks (□) represent the means of the overall scores of pre- and post-OSCEs for the respective groups.

At baseline, there was no statistical difference between the control and intervention groups (*p* > 0.05; Figure 8). Both the intervention and control groups demonstrated a significant improvement in their performance after the OSCE training (intervention group: *p* < 0.05; control group: *p* < 0.05; Figure 8). Furthermore, the intervention group improved significantly in their overall checklist scores compared to the control group (*p* < 0.05; Figure 6; Appendix B; Table A1).

**Figure 9:**
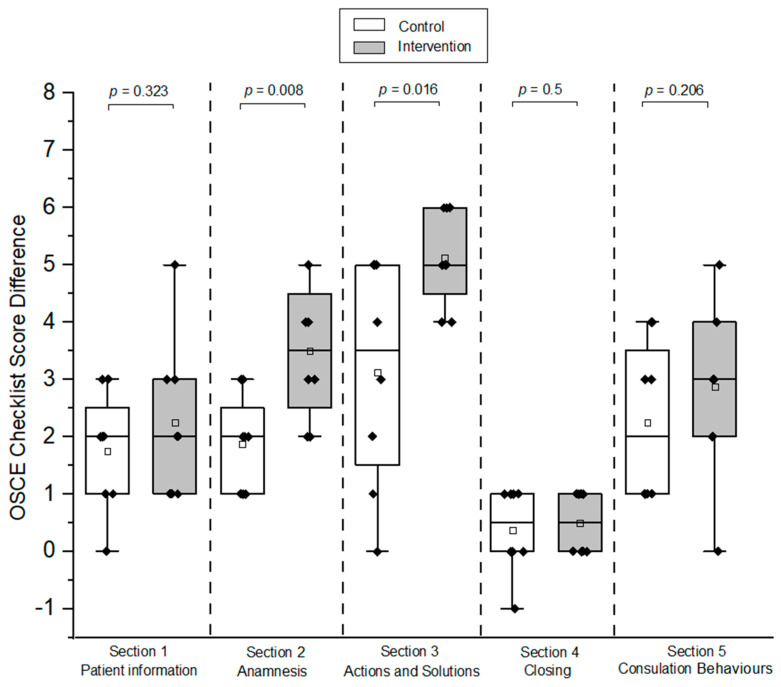
Box plots of OSCE checklist score differences for each section between pre- and post-OSCE for intervention and control groups (HFS-pediatric). A one-sided Mann–Whitney test with a significance level of alpha = 0.05 was used to compare OSCE scores between the respective groups. The black diamonds (♦) indicate the score difference of each participant of post- and pre-OSCE. OSCE = Objective Structured Clinical Examination. HFS = high-fidelity simulator. The hollow blocks (□) represent the means of score differences in each OSCE checklist section for the respective groups.

The intervention group showed a significant improvement in Sections 2 and 3 in comparison to the control group, as shown in Figure 9 (*p* < 0.05), but there was no significant difference for Sections 1, 4, and 5, which are related to patient information, consultation closing, and consultation behaviors, respectively (*p* = 0.323 for Section 1; *p* = 0.5 for Section 4; *p* = 0.206 for Section 5; Figure 9).

## 4. Self-Assessment Questionnaire

### 4.1. Intervention and Control Groups (HFS-Adult)

**Figure 10:**
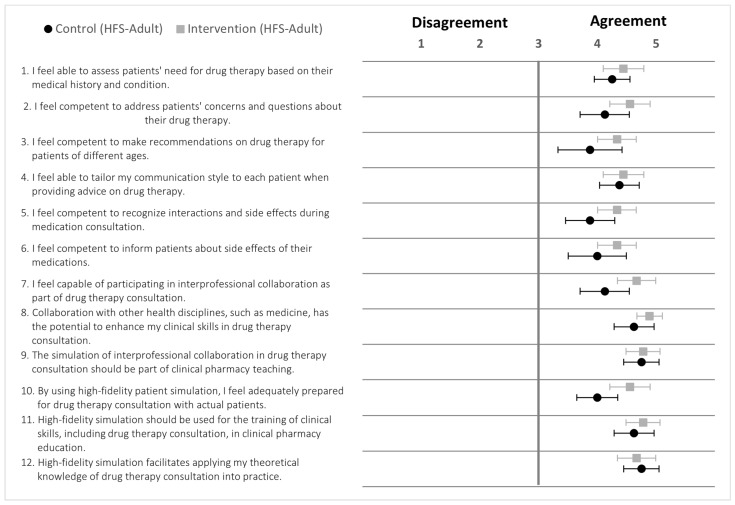
Forest plot of mean values with 95% confidence interval of self-assessment scores for statement 1 to 12 post-OSCES (5-point Likert scale). Black dots (•) = control (HFS-Adult); grey square (■) = intervention (HFS-Adult); *n* = 17. HFS = high-fidelity simulator.

Pharmacy participants in the intervention and control groups showed agreement in self-assessment statements (Figure 10). At baseline, the intervention and control groups demonstrated no significant differences (*p* = 0.115). The post-OSCEs total self-assessment scores between the respective groups demonstrated a significant difference (*p* = 0.015). Additionally, there was a significant difference (*p* < 0.05) between the pre- and post-OSCE total self-assessment scores in the intervention and control groups (Appendix C; Table A2).

### 4.2. Intervention and Control Groups (HFS-Paediatric)

**Figure 11:**
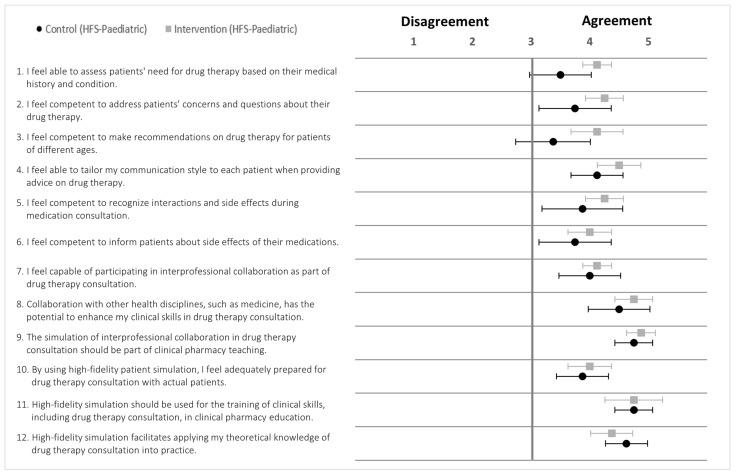
Forest plot of mean values with 95% confidence interval of self-assessment scores for statement 1 to 12 post-OSCES (5-point Likert scale). Black dots (•) = control (HFS-pediatric); grey square (■) = intervention (HFS-pediatric); *n* = 16. HFS = high-fidelity simulator.

At baseline pre-OSCE, the intervention and control groups showed no significant differences in self-assessment scores (*p* = 0.309). The post-OSCEs total self-assessment scores between respective groups revealed significant differences (*p* = 0.042; Figure 11). Moreover, there was a significant difference (*p* < 0.05) between the pre-and post-OSCE total self-assessment scores in both the intervention and control groups (Appendix C; Table A3).

The means of the control group responses for statements 1 and 3 are close to neutral; the confidence interval especially crosses the middle point (neutral = 3), and it shows relatively more variability than intervention group; these statements relate to the pharmacy students’ competency in managing drug therapy for patients of different ages and their ability to assess patients’ need for drug therapy (Figure 11).

## 5. Attitude Survey toward Interprofessional Collaboration

### 5.1. Intervention and Control Groups (HFS-Adult)

**Figure 12:**
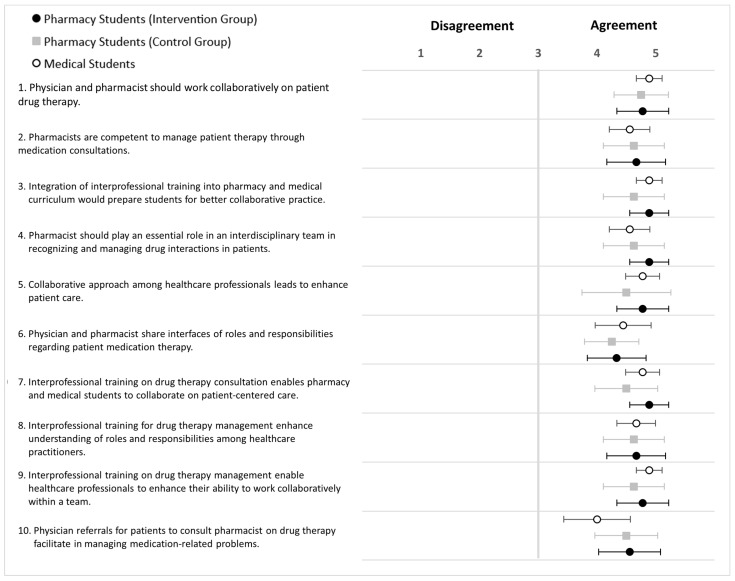
Forest plot of mean values with 95% confidence interval of attitudes scores (HFS-Adult) for statement 1 to 10 after training (5-point Likert scale). Black dots (•) = Pharmacy students (intervention group; grey square (■) = Pharmacy students (control group); white dots (∘) = medical students. Pharmacy students *n* = 17, medical students *n* = 9.

The results of the attitude survey revealed positive responses across medical students and pharmacy students in both study groups (Figure 12; Appendix C; Table A4). However, the responses of medical students were more positive for S1, S3, and S9, which relate to collaborative drug therapy management, the integration of interprofessional training into the curriculum, and enhancing healthcare professionals’ abilities through IPE and collaborative teamwork.

### 5.2. Intervention and Control Groups (HFS-Paediatric)

**Figure 13:**
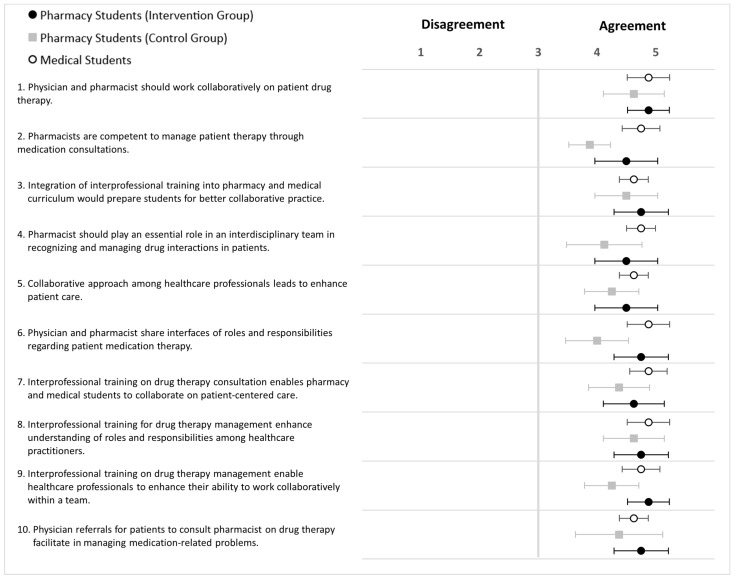
Forest plot of mean values with 95% confidence interval of attitudes scores (HFS-paediatric) for statement 1 to 10 after training (5-point Likert scale). Black dots (•) = Pharmacy students (intervention group; grey square (■) = Pharmacy students (control group); white dots (∘) = medical students. Pharmacy students *n* = 16, medical students *n* = 8.

The survey results showed positive attitudes among the study groups (Figure 13). For certain statements, such as S2, S4, S6, and S7, the medical students tended to be more positive than the pharmacy students in both study groups. These statements related to pharmacy competency, the role of the pharmacist in an interdisciplinary team, sharing of responsibilities between physicians and pharmacists, and the importance of interprofessional training for drug therapy consultation. The overall responses of pharmacy students in the intervention group showed more agreement than those in the control group (Appendix C; Table A5).

## 6. Discussion

In this study, we found that pharmacy students who received interprofessional training performed better in medication therapy consultations compared to those who underwent mono-professional training. Specifically, the overall OSCE performance of pharmacy students in the interprofessional groups for HFS with the adult and pediatric simulator was significantly better than the performance of those in mono-professional groups. Furthermore, the pharmacy students in the interprofessional group performed better in the checklist sections on anamnesis; actions and solutions for the medication-related problems; and consultation behaviors. The self-assessment of pharmacy students in interprofessional groups regarding medication therapy consultation was significantly better in consultation and communication than those in mono-professional groups. This difference was observed for both HFS-adult and -pediatric. Moreover, there was a generally positive attitude towards interprofessional learning among both medical and pharmacy students in both study groups.

In this randomized controlled trial, it was evident that interprofessional training with medical students using adult and pediatric HFS can more efficiently train the medication consultation skills of future pharmacists. In this study, although pharmacy students who received mono-professional training using HFS-adult and -pediatric showed an increase in performance from pre- to post-OSCE, their level of performance did not increase as much as that of those who received interprofessional training. The results of this study support the findings of several studies that have reported improved students’ performance through interprofessional training among medical, nursing, and pharmacy students [36,38,48,63]. However, these previous studies have not sufficiently assessed the impact of IPE specifically on pharmacy students in interprofessional groups compared to those in mono-professional groups. These studies used observational or quasi-experimental designs, as well as RCTs involving control groups consisting of mixed health professional students or single discipline groups, excluding pharmacy.

Our study provides evidence that interprofessional training is superior to mono-professional training for pharmacy students in terms of medication therapy consultation. Furthermore, several studies in Europe have shown the impact of interprofessional simulation learning on health profession students, such as those conducted in the UK [63,64]. For the first time in Germany, we have introduced an interprofessional simulation training model for medical and pharmacy students’ collaboration. This model includes OSCE for the objective assessment of medication consultation skills and utilizes HFS of different age groups (adult and pediatric) to simulate realistic medication care consultations. Therefore, altogether with the results of other studies and the strong evidence from our study, we recommend that interprofessional simulation learning be integrated into the curriculum to train medication consultation skills.

The HFS method showed effectiveness resulting in increased participants’ performance. This could be attributed to the findings that HFS offer a patient-centered experience and allows to enhance clinical skills in a controlled environment [40,65]. In addition, Tokunaga and colleagues demonstrated that human patient simulators improved pharmacy students’ ability to monitor vital signs to identify treatment effects and adverse events [66]. Other studies on implementing interprofessional simulation training for pharmacy students revealed that their performance, attitudes, and communication skills were improved [19,67]. For instance, Kelsch et al. used high-fidelity simulator to improve pharmacy students’ skills and attitudes in working with medical students within interprofessional training [39]. Similarly, Bolesta and Chmil conducted simulated scenarios between pharmacy and nursing students using HFS to improve collaboration skills [38]. However, these studies are not fully comparable to our study as they have used only one type of high-fidelity simulator, such as an adult simulator, rather than different simulators age groups.

During the literature review, it was noted that existing research on interprofessional medication consultation training in Germany, such as the study by Gehrke-Beck et al., did not objectively evaluate the students’ performance [8]. However, our study employed an objective assessment checklist as part of the OSCE to evaluate the performance of the pharmacy participants after mono- and interprofessional training. This focus on using OSCE allowed us to train and evaluate the participants’ medication consultation and collaborative skills through acute cases involving medication-related problems. Given the importance of assessing students’ current level of competence through OSCEs to evaluate and teach interprofessional skills for the design of interprofessional course [41].

By assessing the sections of the OSCE checklist in our study, we identified the sections where interprofessional training using HFS in the medication therapy consultation was more beneficial. Specifically, in HFS-adult sections of anamnesis, which involves the participant gathering the patient’s medical history, as well as actions and solutions for the medication-related problems, and consultation behaviors. Similarly, in the HFS-pediatric, interprofessional training proved useful for sections on anamnesis and action and solutions for medication-related issues. This highlights the effectiveness of the interprofessional training with medical students, particularly on pharmacy students’ performance in taking patient history and problem-solving across patient age groups. While section related to consultation behaviors were not significantly different between the study groups. This could be attributed to the limited practical interaction of the pharmacy students have with the pediatric patient age group during their education in Germany, as demonstrated by Yıldırım Sarı et al., who found that students received HFS training for pediatric practices exhibited more clinical stress compared to those underwent traditional training [68]. Additionally, Lee Chin et al. found that some students exhibited nervousness during simulation sessions, which might have affected their confidence levels [69]. On the other hand, sections such as patient information and closing of the consultation were not affected in the HFS-adult and -pediatric, since both sections involve basic tasks that may not significantly differ based on training type. Future research could explore the long-term impact of interprofessional simulation training on pharmacy students’ real-world performance and evaluate additional training focused on improving consultation behaviors with pediatric patients.

Interprofessional and simulation learning is generally accepted among pharmacy participants’ study groups in the results of the self-assessment questionnaire. Pharmacy students’ responses in HFS-adult and -pediatric for statements that were related to collaborative and HFS learning for medication therapy consultation showed general agreement among participants after the study trainings. It should be noted that despite the improved performances in the mono-professional group within HFS-pediatric, certain pharmacy students showed varied responses, particularly in statements regarding their ability to assess patients’ need for drug therapy based on medical history, as well as related to their competency in managing drug therapy for patients of different ages. The variability in responses could be attributed firstly to the traditional mono-professional method of training commonly used in clinical pharmacy education in Germany, and secondly to the students’ limited practical interaction with pediatric simulators, as demonstrated by some studies that the students could reveal nervousness and clinical stress during simulation sessions [69], especially during pediatric practices [68]. Additionally, HFS training offers practical experience to make real-time decisions that mimics the real-world environment [70], and the pharmacy participants had limited experience with during their education, which also likely contributed to the observed variance in student responses within HFS-pediatric.

In this study, we explored the attitudes of students towards interprofessional collaboration through a post-training survey. The survey statements were generalized and focused on aspects such as collaborative medication management, as well as roles and responsibilities within an interprofessional team. Positive responses were observed among medical and pharmacy participants in both inter- and mono-professional groups. Our findings suggest that the training sessions using HFS for adult and pediatric cases could be attributed to the positive attitude towards interprofessional learning among both medical and pharmacy students. Similarly, Beichler et al. confirmed a significant improvement in attitudes towards interprofessional learning after HFS training [71].

Our study may have some limitations. Firstly, the faculty members, who simulated an adult patient using the simulator and acted as a family member of the pediatric patient were pharmacists and not professional actors. However, those faculty members were not the examiners who filled out the checklist to avoid any possible bias; furthermore, they were instructed and trained through roleplay patient scenarios for all cases to strictly follow the written patient scenario guide and script for each case. The roleplay patient scenarios and written script provided detailed instructions and background information, including patient medical history and additional individual case notes, and outlined the responses that faculty members as actors should deliver during the simulation. Secondly, the pharmacy participants’ performance in the pre- and post-OSCE for HFS-adult and pediatric cases was assessed by OSCE examiners. To avoid examiner-related scoring variations, particularly in the “consultation behaviors” section, both OSCE examiners received detailed instruction and training on the content and grading of all checklist items for both adult and pediatric cases. The individual items in the assessment checklists for HFS-adult and pediatric cases were clearly defined to minimize any potential misinterpretation by the OSCE examiner during assessment. Thirdly, despite randomization, at baseline, the mono-professional group within HFS-adult had significantly different OSCE scores from the interprofessional group. However, the *p*-value (0.046) between the two groups is close to the significance threshold (0.05), which could indicate the sensitivity of the small sample size to Mann–Whitney test. Moreover, further studies with a larger number of participants involving health professions students from multiple disciplines in various universities are recommended. Moreover, the interprofessional training courses using HFS can be challenging due to high costs incurred and time constraints. However, collaboration between university departments or even the presence of a national framework can address these issues. This could provide different interprofessional learning strategies across universities.

## 7. Conclusions

The role of pharmacists in interprofessional collaboration is becoming increasingly significant and recognized in Germany and worldwide. HFS-interprofessional training involving pharmacy and medical students proved to be more effective than HFS-mono-professional training in preparing pharmacy students for medication consultation skills. Specifically, it could be revealed that taking a patient’s medical history and managing medication-related problems can be effectively trained through the integration of interprofessional simulation course into the pharmacy curriculum.

## Figures and Tables

**Table 1:** Characteristics of pharmacy students.

Pharmacy Participants		HFS-Adult (*n* = 17)		HFS-Pediatric (*n* = 16)	
		Intervention (*n* = 9)	Control (*n* = 8)	Intervention (*n* = 8)	Control (*n* = 8)
Age	Mean (±SD)	24 (±2.205)	25 (±3.615)	25 (±4.062)	25 (±2.268)
	Median	23	23	23.5	24
	Range	21–28	22–32	22–34	22–29
Gender	Male, *n* (%)	1 (11%)	2 (25%)	3 (37%)	0 (0%)
	Female, *n* (%)	8 (89%)	6 (75%)	5 (63%)	8 (100%)
Experience in drug therapy consultation (e.g., outpatient or inpatient hospitals)					
Yes, *n* (%)		0 (0%)	1 (12%)	1 (12%)	0 (0%)
No, *n* (%)		9 (100%)	7 (88%)	7 (88%)	8 (100%)

**Table 2:** Characteristics of medical students.

Medical Participants		HFS-Adult (*n* = 9)	HFS-Pediatric (*n* = 8)
Age	Mean (±SD)	24 (±2.550)	23 (±0.518)
	Median	23	23.5
	Range	22–29	22–23
Gender	Male, *n* (%)	5 (56%)	2 (25%)
	Female, *n* (%)	4 (44%)	6 (75%)
Experience in drug therapy consultation (e.g., outpatient or inpatient hospitals)			
Yes, *n* (%)		6 (67%)	3 (37%)
No, *n* (%)		3 (33%)	5 (63%)

## Data Availability

The dataset presented in this study is available from the corresponding author on reasonable request.

## Appendix A. OSCE Checklist

	**OSCE Checklist**			
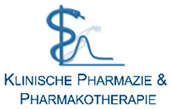
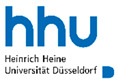
Participant code:			Date:	
(A) **Patient information** ✓ “fulfilled” ✕ “not fulfilled”				Comments
A.1 The participant introduces him/herself to the patient by name and role.				
A2 Confirms patient’s identity (name, date of birth)				
A2.1 The participant asks the patient to provide his/her surname and first name.				
A2.2 The participant asks the patient to provide his/her date of birth to ensure accurate identification.				
A3 Discusses the purpose of the consultation.				
A3.1 The participant explains the purpose of the drug therapy consultation to the patient.				
A3.2 The participant gains the patient’s consent to proceed with the drug therapy consultation.				
(B) **Anamnesis** ✓ “fulfilled” ✕ “not fulfilled”				Comments
**B1 History of Present Illness**				
B1.1 The participant asks for further information about the patient’s main concern.				
B1.2 The participant asks for further information about the timing of the symptoms (onset and duration).				
B1.3 The participant asks about any associated symptoms that the patient has noticed.				
B1.4 The participant assesses the patient’s adherence to current medications.				
B1.5 The participant asks about any prescribed or over-the-counter medications that the patient is currently taking, which may not be listed in the patient sheet.				
**B2 Lab Values/Vital signs Interpretation**				
B2.1 The participant assesses any abnormal lab values that may indicate risk.				
B2.2 The participant assesses the patient’s vital signs.				
**B3 Personal and Social History** (**the participant asks for further information about only one point depending on the OSCE case, other points are mentioned in the patient information sheet**)				
B3.1 ask for information about tobacco.				
B3.2 ask for information about Alcohol.				
B3.3 ask for information about usual dietary practices.				
B3.4 ask for information about current physical activity.				
B3.5 ask for information about trafficability.				
(C) **Actions & Solutions** ✓ “fulfilled” ✕ “not fulfilled”				Comments
**C1 Drug Interactions and Side Effects**				
C1.1 The participant identifies possible drug–drug interactions in the patient’s medications.				
C1.2 The participant provides optimization recommendations to possible drug–drug interactions related to point C1.1.				
C1.3 The participant recognises possible drug-related problem.				
C1.4 The participant explains the reasons for occurrence of the drug-related problem related to point C1.3.				
C1.5 The participant provides optimisation recommendations related to point C1.3.				
**C2 Administration Timing/Technique and Side Effects**:				
C2.1 The participant asks about the patient’s understanding of the appropriate administration timing or technique for a particular medication.				
C2.2 The participant recognises inappropriate administration timing or technique related to point C2.1.				
C2.3 The participant explains the reasons for the side effects that occurred due the identified inappropriate administration timing/technique.				
C2.4 The participant provides optimisation recommendations related to inappropriate timing or technique related to point C2.1.				
C2.5 The participant provides further recommendations related to administration timing/technique depend on each case.				
(D) **Closing** ✓ “fulfilled” ✕ “not fulfilled”				Comments
D.1 The participant instructs the patient to report his/her medications (including over-the-counter products/food supplements) to the physician.				
D.2 The participant provides opportunity to the patient to ask any further questions.				
D3. The participant expresses gratitude for the patient’s collaboration and asks the patient to follow up.				
(E) **Consultation Behaviors** ✓ “fulfilled” ✕ “not fulfilled”				Comments
E.1 The participant listens actively and allows the patient to complete statements.				
E.2 The participant uses open and closed questions when appropriate.				
E.3 The participant responds effectively to the patient’s questions and concerns in informative and reassuring way.				
E.4 The participant shows support and respond to the patient’s feelings (angry/frustrated) and needs.				
E.5 The participant avoids scientific words (unnecessary jargon) and explain medical concepts in patient-friendly terms.				
E.6 The participant demonstrates confidence during the consultation, avoiding moments of silence and confusion.				
E.7 The participant maintains control over the drug therapy consultation and avoids irrelevant discussions.				
E.8 The participant employs effective non-verbal communication including appropriate gestures and friendly facial expressions.				
E.9 The participant manages the OSCE time effectively and ensures that the drug therapy consultation remains within the designated time frame.				
Name of examiner:				Date:
Signature of examiner:				

## Appendix B

**Table A1:** Pre- and Post OSCE-Scores.

	HFS-Adult		HFS-Pediatric	
	Intervention Mean (SD)	Control Mean (SD)	Intervention Mean (SD)	Control Mean (SD)
Section 1				
Pre-OSCE Score	0.44 (0.53)	0.13 (0.35)	1.00 (0.53)	1.50 (0.53)
Post-OSCE Score	1.89 (1.27)	1.88 (0.83)	3.25 (1.16)	3.25 (0.71)
Score Difference	1.44 (1.33)	1.75 (0.89)	2.25 (1.39)	1.75 (1.04)
*p*-Value	0.818		0.323	
Section 2				
Pre-OSCE Score	1.56 (0.73)	2.00 (1.07)	1.38 (1.19)	2.25 (0.89)
Post-OSCE Score	4.78 (0.97)	3.38 (0.92)	4.88 (0.83)	4.13 (1.25)
Score Difference	3.22 (1.09)	1.38 (0.74)	3.50 (1.20)	1.88 (0.83)
*p*-Value	0.002		0.008	
Section 3				
Pre-OSCE Score	1.00 (0.87)	2.88 (1.13)	1.00 (0.76)	1.25 (1.04)
Post-OSCE Score	6.44 (2.13)	5.38 (2.07)	6.13 (1.25)	4.38 (1.51)
Score Difference	5.44 (2.30)	2.50 (1.93)	5.13 (0.83)	3.13 (1.96)
*p*-Value	0.008		0.016	
Section 4				
Pre-OSCE Score	1.11 (0.60)	0.75 (0.71)	0.75 (0.46)	1.00 (0.53)
Post-OSCE Score	2.00 (0.71)	1.63 (0.74)	1.25 (0.46)	1.38 (0.74)
Score Difference	0.89 (0.60)	0.88 (0.64)	0.50 (0.53)	0.38 (0.74)
*p*-Value	0.583		0.5	
Section 5				
Pre-OSCE Score	5.78 (0.97)	6.25 (1.04)	4.88 (1.73)	5.38 (1.41)
Post-OSCE Score	8.11 (0.60)	7.25 (1.49)	7.75 (0.46)	7.75 (0.46)
Score Difference	2.33 (1.12)	0.88 (0.83)	2.88 (1.55)	2.25 (1.39)
*p*-Value	0.009		0.206	
Total				
Pre-OSCE Score	9.89 (1.27)	12.00 (2.20)	9.00 (2.27)	11.38 (2.56)
Post-OSCE Score	23.22 (2.22)	19.50 (4.41)	23.25 (1.28)	20.88 (3.27)
Score Difference	13.33 (2.40)	7.38 (3.29)	14.25 (2.38)	9.38 (2.20)
*p*-Value	0.001		0.002	

## Appendix C

**Table A2:** Overall Self-Assessment Survey scores of pharmacy students in control and intervention groups (HFS-adult).

Group	Pre-Training	Post-Training
	Mean	Mean
	(CI)	(CI)
**Intervention**	41.22	54.78
(*n* = 9)	(3.18)	(1.38)
**Control**	46.38	51.38
(*n* = 8)	(3.68)	(2.77)

**Table A3:** Overall Self-Assessment Survey scores of pharmacy students in control and intervention groups (HFS-pediatric).

Group	Pre-Training	Post-Training
	Mean	Mean
	(CI)	(CI)
**Intervention**	43.38	52.13
(*n* = 8)	(2.64)	(2.38)
**Control**	40.63	48.88
(*n* = 8)	(3.87)	(3.00)

**Table A4:** Overall Attitudes Survey Scores across medical students and pharmacy students in the control and intervention groups (HFS-Adult) after Training.

Group	Intervention (Pharmacy Students) (*n* = 9)	Intervention (Medical Students) (*n* = 9)	Control (Pharmacy Students) (*n* = 8)
**Mean**	47.22	46.44	45.63
(**CI**)	(1.75)	(1.39)	(2.09)

**Table A5:** Average Overall Attitudes Survey Scores across medical students and pharmacy students in the control and intervention groups (HFS-pediatric).

Group	Intervention (Pharmacy Students) (*n* = 8)	Intervention (Medical Students) (*n* = 8)	Control (Pharmacy Students) (*n* = 8)
**Mean**	47.00	48.00	43.38
(**CI**)	(2.19)	(1.57)	(2.67)
